# SESN1 is a FOXO3 effector that counteracts human skeletal muscle ageing

**DOI:** 10.1111/cpr.13455

**Published:** 2023-05-17

**Authors:** Ying Jing, Yuesheng Zuo, Liang Sun, Zheng‐Rong Yu, Shuai Ma, Huifang Hu, Qian Zhao, Daoyuan Huang, Weiqi Zhang, Juan Carlos Izpisua Belmonte, Yang Yu, Jing Qu, Guang‐Hui Liu, Si Wang

**Affiliations:** ^1^ State Key Laboratory of Stem Cell and Reproductive Biology, Institute of Zoology Chinese Academy of Sciences Beijing China; ^2^ University of Chinese Academy of Sciences Beijing China; ^3^ Advanced Innovation Center for Human Brain Protection, National Clinical Research Center for Geriatric Disorders Xuanwu Hospital Capital Medical University Beijing China; ^4^ Aging Translational Medicine Center, International Center for Aging and Cancer, Beijing Municipal Geriatric Medical Research Center, Xuanwu Hospital Capital Medical University Beijing China; ^5^ CAS Key Laboratory of Genomic and Precision Medicine, Beijing Institute of Genomics Chinese Academy of Sciences Beijing China; ^6^ China National Center for Bioinformation Beijing China; ^7^ The NHC Key Laboratory of Geriatrics, Institute of Geriatric Medicine, Chinese Academy of Medical Sciences Beijing Hospital/National Center of Gerontology of National Health Commission Beijing China; ^8^ The NHC Key Laboratory of Drug Addiction Medicine Kunming Medical University Kunming Yunnan China; ^9^ Aging Biomarker Consortium Beijing China; ^10^ Department of Orthopaedics Peking University First Hospital Beijing China; ^11^ State Key Laboratory of Membrane Biology, Institute of Zoology Chinese Academy of Sciences Beijing China; ^12^ Institute for Stem Cell and Regeneration, Chinese Academy of Sciences Beijing China; ^13^ Beijing Institute for Stem Cell and Regenerative Medicine Beijing China; ^14^ Sino‐Danish College University of Chinese Academy of Sciences Beijing China; ^15^ Sino‐Danish Center for Education and Research Beijing China; ^16^ Altos Labs San Diego California USA; ^17^ Department of Obstetrics and Gynecology, Center for Reproductive Medicine Peking University, Third Hospital Beijing China; ^18^ Clinical Stem Cell Research Center Peking University, Third Hospital Beijing China; ^19^ The Fifth People's Hospital of Chongqing Chongqing China

## Abstract

Sarcopenia, a skeletal muscle disorder in which loss of muscle mass and function progresses with age, is associated with increased overall frailty, risk of falling and mortality in the elders. Here, we reveal that SESN1 safeguards skeletal muscle from ageing downstream of the longevity gene *FOXO3*, which we recently reported is a geroprotector in primate skeletal muscle. Knockdown of *SESN1* mimicked the human myotube ageing phenotypes observed in the FOXO3‐deficient human myotubes, whereas genetic activation of SESN1 alleviated human myotube senescence. Of note, SESN1 was identified as a protective secretory factor against muscle atrophy. Administration of recombinant SESN1 protein attenuated senescence of human myotubes in vitro and facilitated muscle regeneration in vivo. Altogether, we unveil a key role of SESN1 downstream of FOXO3 in protecting skeletal muscle from ageing, providing diagnostic biomarkers and intervention strategies for counteracting skeletal muscle ageing and related diseases.

## Graphical abstract

Here, we reveal that SESN1 safeguards skeletal muscle from ageing downstream of the longevity gene *FOXO3*, which we recently reported is a geroprotector in primate skeletal muscle. Notably, SESN1 levels were reduced in the serum of elderly human individuals, implying that SESN1 could function as a potent circulating biomarker to predict progressive skeletal muscle atrophy. More importantly, we propose that recombinant SESN1 protein may have potential as a therapeutic agent capable of attenuating myofibre senescence and promoting muscle regeneration.

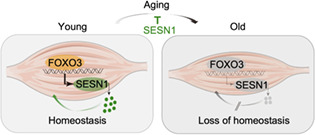

## 1 INTRODUCTION

Skeletal muscle, comprising 30%–40% of total body mass, controls movement and maintains posture by attaching to bones directly or indirectly.^1^, ^2^ By secreting myokines, skeletal muscle exerts systemic effects on the whole body and plays an important role in maintaining the homeostasis and health of the body. However, with ageing, decline in skeletal muscle mass and function, known as sarcopenia, reduces capacity for movement, maintaining balance and other physical functions, increases the risk of falls, disability and even death.^3^ ^,^ ^4^ Therefore, understanding the mechanisms underlying maintenance of skeletal muscle homeostasis and how these are impacted by ageing are of great scientific and clinical significance.

To a limited extent, sarcopenia can be slowed down by physical therapy or nutritional approaches.^5^, ^6^, ^7^ Regular training can improve muscle mass and strength by increasing protein synthesis, myofibrillar count and muscle fibre cross‐sectional area.^8^ Nutrition interventions, through ensuring adequate intake of protein, creatine, vitamin D, Omega‐3 fatty acids and so on, primarily maintain muscle mass.^9^, ^10^ In general, the molecular basis of skeletal muscle ageing has been challenging to pin down, and as a result, progress towards the development of intervention therapies has been slow. Nevertheless, identifying and validating the regulatory mechanism of skeletal muscle ageing remains an important goal for the development of future effective and evidence‐based intervention measures for skeletal muscle ageing.

Sestrins (Sesns) are a family of highly conserved and ubiquitous metabolic proteins that are induced in cells in response to environmental stresses, including oxidative stress, DNA damage, hypoxia and starvation.^11^, ^12^, ^13^ Previous studies have shown that Sesns can play protective roles in various diseases such as metabolic disorders, lipid accumulation and insulin resistance.^14^ Vertebrates express three different types of Sesns, namely SESN1, SESN2 and SESN3.^15^ SESN1, the earliest known protein in the Sestrins family, also known as PA26, has been identified as a target of the tumour‐suppressing protein p53.^16^ Although SESN1 has been reported to have multiple effects on maintaining physiological homeostasis, to our knowledge, it has not previously been linked to primate skeletal muscle ageing.

In this study, we identified and experimentally validated that downregulation of the FOXO3‐SESN1 axis is a mechanism that triggers skeletal muscle ageing in primates. An important finding with clinical implications is that genetic activation of endogenous SESN1 or pharmacological treatment with recombinant SESN1 protein counteracted the onset of human myotube senescence in vitro and enabled skeletal muscle regeneration *in vivo*. Our study strengthens the understanding about the mechanistic underpinning of primate skeletal muscle ageing and facilitates the development of novel therapeutical intervention strategies to combat age‐associated muscle degenerative disorders.

## 2 MATERIALS AND METHODS

### 2.1 Ethical statement

This study was conducted in accordance with ethical treatment guidelines for animals and was approved by the Animal Care and Use Committee of the Institute of Zoology, Chinese Academy of Sciences. All animals and cells used in this study have been approved by the Ethics Review Committee of the Institute of Zoology of the Chinese Academy of Sciences. The collection and use of human serum in this study were obtained from Biobank under the approval given by the Research Ethics committee of the Beijing Hospital. The collection and use of human skeletal muscle in this study were performed under the approval of the Research Ethics committee of the Peking University First Hospital.

### 2.2 Experimental animals

C57BL/6J mice (16 months old) were purchased from SPF Biotechnology Co., Ltd and housed conventionally in a constant temperature (25°C) and humidity (50%–60%) animal room, with a 12 h light–dark cycle and free access to normal diet and water.

### 2.3 Human serum sampling

Human serum samples from healthy young (18–25 years old, *n* = 40) and old (65–80 years old, *n* = 40) donors were collected from Beijing Hospital.

### 2.4 Tissue sampling

Skeletal muscle tissues were harvested from human and mice as previously described.^17^, ^20^ The skeletal muscle tissues, removed of attached fat or fascia tissues, were fixed in 4% paraformaldehyde (PFA) at 4°C for 24 h and embedded in paraffin or Tissue‐Tek optimum cutting temperature (O.C.T) compound (Sakura Finetek) for the following histological analysis. The remaining tissues were stored in liquid nitrogen for other RNA or protein analyses.

### 2.5 Cell culture

Human embryonic stem cells (hESCs; Line H9, from WiCell Research) were cultured in hESC culture medium on mitomycin C‐inactivated mouse embryonic fibroblasts.^18^ The hESC culture medium contained Dulbecco's Modified Eagle Medium (DMEM)/F12 (Gibco), 20% Knockout Serum Replacement (Gibco), 2 mM GlutaMAX (Gibco), 0.1 mM non‐essential amino acids (Gibco), 55 μM β‐mercaptoethanol (Invitrogen), 10 ng/mL basic fibroblast growth factor (Joint Protein Central) and 1% penicillin/streptomycin (Gibco). Human myotubes (hMyotubes) were cultured in high‐glucose DMEM medium (Hyclone) containing 2% horse serum (Gibco) and 2 mM GlutaMAX (Gibco) at 37°C with 5% CO_2_ and 3%–5% O_2_.^19^, ^20^ HEK293T cells were cultured in high‐glucose DMEM (Hyclone) supplemented with 10% fetal bovine serum (FBS, Gibco) at 37°C with 5% CO_2_. There was no mycoplasma contamination observed during cell culture.

### 2.6 Cardiotoxin injury‐induced skeletal muscle damage in mice

Skeletal muscle injury was performed as described previously.^17^ Briefly, C57BL/6J male mice (16 months old) were randomly divided into an uninjured group treated with phosphate buffered saline (PBS) (referred to Sham group), and the injured group treated with PBS (referred to post‐injury‐Vehicle group) or recombinant SESN1 protein (referred to post‐injury‐rSESN1 group). For the injured group, 10 μM of cardiotoxin (CTX, Latoxan) in 25 or 50 μL PBS was injected twice into tibialis anterior muscle or quadriceps muscle, respectively. In the following 7 days, recombinant SESN1 protein (0.5 μg/mL) in 30 or 60 μL PBS was injected into tibialis anterior muscle or quadriceps muscle, respectively.

### 2.7 Physical function measurements in mice

#### 2.7.1 Grip strength test

Forelimb and hind limb grip strength was measured using a grip strength meter (Panlab Grid Strength Meter, LE902) as previously reported.^21^, ^22^ Briefly, mice in Sham, post‐injury‐Vehicle and post‐injury‐rSESN1 groups were held by the tail and allowed to hold the grid of the apparatus. Mice were gently pulled away from the grid until they released the grid. The peak pull force was measured 10 times at 1‐min interval for each test, and the mean of the values was recorded as the grip strength of each mouse.

#### 2.7.2 Rotarod test

Motor coordination was assessed in mice in Sham, post‐injury‐Vehicle and post‐injury‐rSESN1 groups with a rotarod test, performed on a rotating rod (Yiyan Tech, YLS‐4C) that accelerated from 4 to 44 rpm/min with an acceleration of 8 rpm/min approximately per minute. The time spent on the rod (before falling) per trial was recorded. Animals were trained three trials per day for 3 consecutive days before formal experiment, with measurements at 5‐min intervals. For each mouse, the average time from start of each trial to dropping down was recorded.

#### 2.7.3 Treadmill performance test

Mice were trained on a treadmill (SANS Bio Instrument, SA101) at a 5° incline with an electrical stimulation (2 mA) over 3 days before formal experiments. Each trial sustained for 20 min with the speed accelerated from 5 to 20 m/min. On the test day, the mice ran on the treadmill with an acceleration of 2 rpm/min. The maximal speed, time and distance to exhaustion were recorded when the mice were unable to return to the treadmill and stayed on the electrode for more than 10 s.

### 2.8 Immunofluorescence staining

Immunofluorescence staining was performed as previously described with slight modifications.^23^ For tissues, the muscle tissue embedded in the O.C.T. were cut into 10 μm cryosections by the Leica CM3050S cryostat. The cryosections were air‐dried for 15 min and washed three times in PBS before fixed with 4% PFA for another 20 min. Next, these sections were permeabilized with 0.4% Triton X‐100 in PBS for 1 h, and again rinsed with PBS three times. Cells were fixed with 4% PFA for 20 min and rinsed with PBS twice, and permeabilized with 0.4% Triton X‐100 (Sigma‐Aldrich) for 1 h at room temperature (RT). After blocking for 1 h at RT, the sections or cells were incubated with primary antibodies overnight at 4°C. Subsequently, the samples were washed several times with PBS and incubated with fluorescently labelled secondary antibodies for 1 h at RT. The nuclei were stained with Hoechst 33342 (Thermo Fisher Scientific), and washed three times in PBS and then mounted in VECTERSHIELD anti‐fading mounting medium (Vector Laboratories). The image was acquired using a confocal laser scanning microscope (Leica TCS SP5 II). The antibodies used for immunofluorescence analysis are listed in Table S3.

### 2.9 Lentivirus packaging

HEK293T cells were used for lentivirus packaging. After co‐transfecting with lentiviral vectors, and packaging vectors psPAX2 (Addgene) and pMD2.G (Addgene) using Lipofectamine 3000 Transfection Reagent (Thermo Fisher Scientific) for 48 and 72 h, cell‐conditioned medium of HEK293T cells were collected and ultracentrifugation at 19,400 *g* at 4°C for 2.5 h. Viral particles as pellets were then suspended in MEMα medium for further application.

### 2.10 Knockdown of *SESN1* using small interfering RNA

To knockdown *SESN1* expression, we transfected corresponding small interfering RNAs (siRNAs) or nontarget siRNAs into hMyotubes using RNAiMAX. Briefly, 25 μM of negative control duplex (si‐NC) or siRNAs against *SESN1* (si‐*SESN1*) was mixed with 100 μL of Opti‐MEM (Gibco) and 2 μL Lipofectamine™ RNAiMAX Transfection Reagent (Thermo Fisher Scientific) and then added to one well of 12‐well plates. The culture medium was replaced with fresh medium after an 8‐h incubation, and the cells were collected for analysis 72 h after transfection. *SESN1* siRNAs were synthesized by RiboBio (China), and siRNA sequences are listed in Table S4.

### 2.11 Activation of endogenous expression of SESN1 using CRISPR/dCas9 transcriptional activation system

The CRISPR/dCas9‐mediated gene activation system was performed as previously described.^18^ Briefly, guide RNAs, designed to target the *SESN1* locus at the transcription start site (TSS), and two non‐targeting controls (NTCs) were constructed into lentiSAMv2 vector (Addgene). For the induction of endogenous expression of SESN1, *FOXO3* ^*−/−*^ human myotube progenitor cells were co‐transduced with Lenti‐SAMv2 and Lenti‐MPHv2 (Addgene) to transcriptionally activate SESN1 expression for 48 h following blasticidin and hygromycin selection for 6 days. The selected cells were then differentiated into hMyotubes and collected for the subsequent analyses.

### 2.12 Treatment of hMyotubes with recombinant SESN1 protein

*FOXO3* ^*−/−*^ hMyotubes were treated with 0.5 μg/mL recombinant SESN1 protein (Abcam); and culture medium, including recombinant SESN1 protein, was changed every other day. And 96 h later, cells were quickly washed with PBS and collected for the subsequent analyses.

### 2.13 Senescence‐associated β‐galactosidase staining

Senescence‐associated β‐galactosidase (SA‐β‐gal) staining of hMyotube was conducted as previously described.^24^ Briefly, cells were fixed in 2% formaldehyde and 0.2% glutaraldehyde at RT for 5 min. Then, fixed cells were stained with fresh staining solution containing X‐gal at 37°C overnight after washing twice with PBS. Fields of view were randomly selected in each well, and the percentages of SA‐β‐gal‐positive cells was counted by ImageJ software.

### 2.14 RNA isolation and real‐time quantitative PCR

Total RNA was extracted using TRIzol Reagent (Life Technologies) according to the manufacturer's protocol. Then, the GoScript™ Reverse Transcription System (Promega) was used to reverse transcribe the cDNA. And samples were used for real‐time quantitative PCR (RT‐qPCR) assay with THUNDERBIRD SYBR qPCR Mix (Toyobo) on a CFX384 Real‐Time PCR system (Bio‐Rad), and statistical significances were assessed by an independent‐sample *t* test. The primer pairs used in this study are listed in Table S4.

### 2.15 Western blot analysis

Western blot analysis was performed as previously described.^25^, ^26^ Tissues or cells were lysed with SDS lysis buffer containing 2% SDS and 62.5 mM Tris–HCl (pH = 6.8) and incubated at 100°C for 10 min. The protein concentration was quantified using a bicinchoninic acid (BCA) quantification kit. The protein lysates were subjected to SDS‐PAGE and subsequently transferred to a PVDF (polyvinylidene fluoride) membrane (Millipore). After blocking in 5% skimmed milk powder (BBI Life Sciences) in 1× TBST (Tris‐buffered saline with 0.1% Tween 20), the membranes were incubated with the primary antibodies overnight at 4°C and with the HRP‐conjugated secondary antibodies (ZSGB‐BIO) for 1 h at RT, followed by visualization using the ChemiDoc XRS system (Bio‐Rad). The band intensity quantification was performed with ImageJ software. The antibodies used are listed in Table S3.

### 2.16 Chromatin immunoprecipitation‐qPCR

Chromatin immunoprecipitation (ChIP)‐qPCR was performed following the previously published protocol with slight modification.^18^ Briefly, hMyotubes were collected and washed in PBS, then cross‐linked with 1% (vol/vol) formaldehyde diluted in PBS for 12 min. Next, to stop the cross‐linking reaction, cells were incubated in 0.125 M Glycine for 5 min at RT. After washes with PBS, cells were lysed in lysis buffer (50 mM Tris–HCl, 10 mM EDTA, 1% SDS and pH 8.0) for 5 min. The mixture was sonicated by a Bioruptor® Plus device (Diagenode), and supernatants were incubated overnight at 4°C with Protein A/G Dynabeads (Thermo Fisher Scientific) conjugated with 2.4 μg anti‐FOXO3 antibody or rabbit IgG. Subsequently, immunoprecipitated chromatin eluting and decross‐linking were performed at 68°C for 3 h on a thermomixer. DNA was then collected by the phenol‐chloroform‐isoamylalcohol extraction and ethanol precipitation method, after which purified DNA was subjected to qPCR for evaluation of FOXO3 occupation at the promoter of *SESN1* gene. The primers used for ChIP‐qPCR are listed in Table S4. The antibodies used in this study are listed in Table S3.

### 2.17 Luciferase reporter assay

Luciferase reporter assay was performed as previously described.^27^ Partial *SESN1* promoter was amplified by PCR and cloned into the pGL3‐Basic vector (Promega). The mutant version of the pGL3‐SESN1 vector was constructed with Fast Mutagenesis System kit (TransGen Biotech). The pGL3 vectors, together with a Renilla plasmid (Promega), were co‐transfected into human myotube progenitor cells with Lipofectamine 3000. After 48 h, cells were cultured in MEMα culture medium without FBS for 24 h and then lysed for the measurement using the Dual‐Luciferase Assay Kit (Vigorous Biotechnology), and firefly and Renilla luciferase activity were measured with a Synergy H1 Hybrid Reader (Bio‐Tek). Primers were synthesized by Tsingke Biotech. The primers are listed in Table S4.

### 2.18 Enzyme‐linked immunosorbent assay

Protein levels of SESN1 in human serum and hMyotube‐conditioned medium were measured by sandwich enzyme‐linked immunosorbent assay (ELISA) as per the manufacturer's instructions (Fine Test). Protein levels of SESN1 in the hMyotube‐conditioned medium were normalized to the number of nuclei of the hMyotubes. The absorbance of each well was scanned at 450 nm using Synergy H1 Hybrid Reader (Bio‐Tek).

### 2.19 Nuclei isolation and snRNA‐seq on the 10× Genomics platform

Isolation of nuclei was performed following a previously published protocol with some modifications.^28^ Briefly, a piece of frozen mouse gastrocnemius muscle tissues was grinded into powder separately with liquid nitrogen. This power was homogenized in 1.0 mL homogenization buffer containing 250 mM sucrose, 5 mM MgCl_2_, 25 mM KCl, 10 mM Tris buffer, 1 μM DTT, 1× protease inhibitor, 0.4 U/μL RNaseIn, 0.2 U/μL Superasin, 0.1% Triton X‐100, 1 μM propidium iodide (PI) and 10 ng/mL Hoechst 33342 using a freezing multisample tissue grinding system (60 Hz and 30 s per time, four times in total). Samples were filtered through a 40‐μm cell strainer (BD Falcon), centrifuged at 3000 *g* for 8 min at 4°C, and resuspended in PBS supplemented with 0.3% bovine serum albumin, 0.4 U/μL RNaseIn and 0.2 U/μL Superasin. Hoechst 33342 and PI double‐positive nuclei were sorted using fluorescence‐activated cell sorting (BD Influx) and counted with a dual‐fluorescence cell counter (Luna‐FL™, Logos Biosystems). Approximately 7000 nuclei were captured for each sample with 10× Genomics Chromium Single Cell Kit (10× Genomics) version 3 following the standard protocol and then sequenced in a NovaSeq 6000 sequencing system (Illumina, 20012866).

### 2.20 Bulk RNA‐seq library construction and sequencing

RNA quality control, library construction and high‐throughput sequencing were performed for each sample as previously described.^29^ Briefly, sequencing libraries were prepared using NEBNext® UltraTM RNA Library Prep Kit for Illumina® (NEB) and individually indexed. The resultant libraries were sequenced on an Illumina paired‐end sequencing platform by 150‐bp read length by Annoroad Gene Technology Co. Ltd.

### 2.21 Bulk RNA‐seq data processing

RNA‐seq data were processed as previously described.^30^ Briefly, to trim adapter sequence and remove low quality reads, the raw sequencing reads were first processed with Trim Galore (http://www.bioinformatics.babraham.ac.uk/projects/trim_galore/; version 0.6.6). Then, the cleaned reads were mapped against human reference hg19 downloaded from Ensembl^31^ with HISAT2 (version 2.2.1),^32^ and mapped reads counted with HTSeq (version 0.13.5).^33^ Differentially expressed gene (DEG) analysis was conducted with DESeq2 (version 1.28.1)^34^ in R, and DEGs were identified with the cutoff of |Log_2_FC| > 0.5 and adjusted *p* values < 0.05.

### 2.22 Processing and quality control of snRNA‐seq data

Raw sequencing reads of mouse skeletal muscle were aligned to the pre‐mRNA reference (Ensembl, mm10) and counted using Cell Ranger (version 4.0.0) with the default parameters. The raw count matrices were filtered using CellBender (version 0.2.0) software in order to eliminate the contamination of background mRNA.^35^ Seurat (version 3.2.2) object of each sample was constructed from the decontaminated matrix and nuclei with genes fewer than 200 or mitochondrial ratio more than 5% were discarded.^36^ Doublet removal was performed with DoubletFinder (version 2.0.3).^37^ Afterwards, the clusters lacking specific marker genes and with relatively low gene content were also discarded.

### 2.23 Integration, clustering and identification of cell types

Integration, clustering and identification of cell types were processed under the corresponding pipeline of Seurat package. First, count matrix of each sample was normalized using the ‘SCTransform’ function. Features and anchors for downstream integration were selected with corresponding pipeline using the ‘FindIntegrationAnchors’ and ‘IntegrateData’ function, ensuring that calculation was based on all necessary Pearson residuals. After data integration and scaling, principal component analysis (PCA) was performed with the ‘RunPCA’ function, and dataset was then clustered using the ‘FindNeighbors’ and ‘FindClusters’ function. Dimensionality was reduced with the ‘RunUMAP’ function. Cell types were identified according to the expression levels of the classic marker genes. The marker genes of each cell type were calculated using the ‘FindAllMarkers’ function with the cutoff of |LogFC| > 0.5 and adjusted *p* values < 0.05 using *t* test. Marker genes for each cell type are shown in Table S2.

### 2.24 Analysis of DEGs from snRNA‐seq data

DEGs between post‐injury‐Vehicle and Sham groups (upon CTX‐induced injury) and between post‐injury‐rSESN1 and post‐injury‐Vehicle groups (upon rSESN1 protein treatment) in mouse skeletal muscle were analysed by the function of ‘FindMarkers’ in Seurat using Wilcoxon rank‐sum test and were identified with the cutoff of |LogFC| > 0.25 and adjusted *p* values < 0.05. Rescued DEGs were defined as genes exhibiting the opposite changes upon CTX‐induced injury and rSESN1 protein treatment. DEG lists for each cell type are shown in Table S1.

### 2.25 Gene ontology enrichment analysis

Gene ontology (GO) enrichment analysis was performed with Metascape.^38^ Representative terms were selected with the cutoff of *p* values < 0.01 and visualized with ggplot2 R package (version 3.3.2).^39^

### 2.26 Gene set score analysis

Regeneration‐related gene set was acquired from Regeneration Roadmap,^40^ which was used for scoring each input cell with the Seurat function ‘AddModuleScore’. Differences in the scores between post‐injury‐rSESN1 and post‐injury‐Vehicle were analysed using ggpubr R package (version 0.2.4) via the Wilcoxon test.

### 2.27 Analysis of FOXO3 target genes

A transcription factor regulatory network of ageing‐associated DEGs in cynomolgus monkey skeletal muscle was established under SCENIC (Single‐Cell Regulatory Network Inference and Clustering) standard workflow following previous work.^20^, ^41^ At first, the candidate target genes co‐expressed with FOXO3 were identified using Genie3. The weight scores calculated by Genie3 represent the co‐expression levels between FOXO3 and its candidate target genes. Next, each co‐expression module was analysed using RcisTarget^41^ to identify putative direct‐binding target genes of FOXO3. Only target genes with high‐confident motif enrichment were selected for the downstream analysis.

### 2.28 Statistical analyses

All data were statistically analysed using the two‐tailed *t* test or Wilcoxon Rank Sum test to compare differences between different groups, assuming equal variance with PRISM software (GraphPad 8 Software). *p* Values are presented in indicated figures.

## 3 RESULTS

### 3.1 SESN1 acts as a major downstream gene of geroprotector FOXO3 in primate skeletal muscle

**FIGURE 1:**
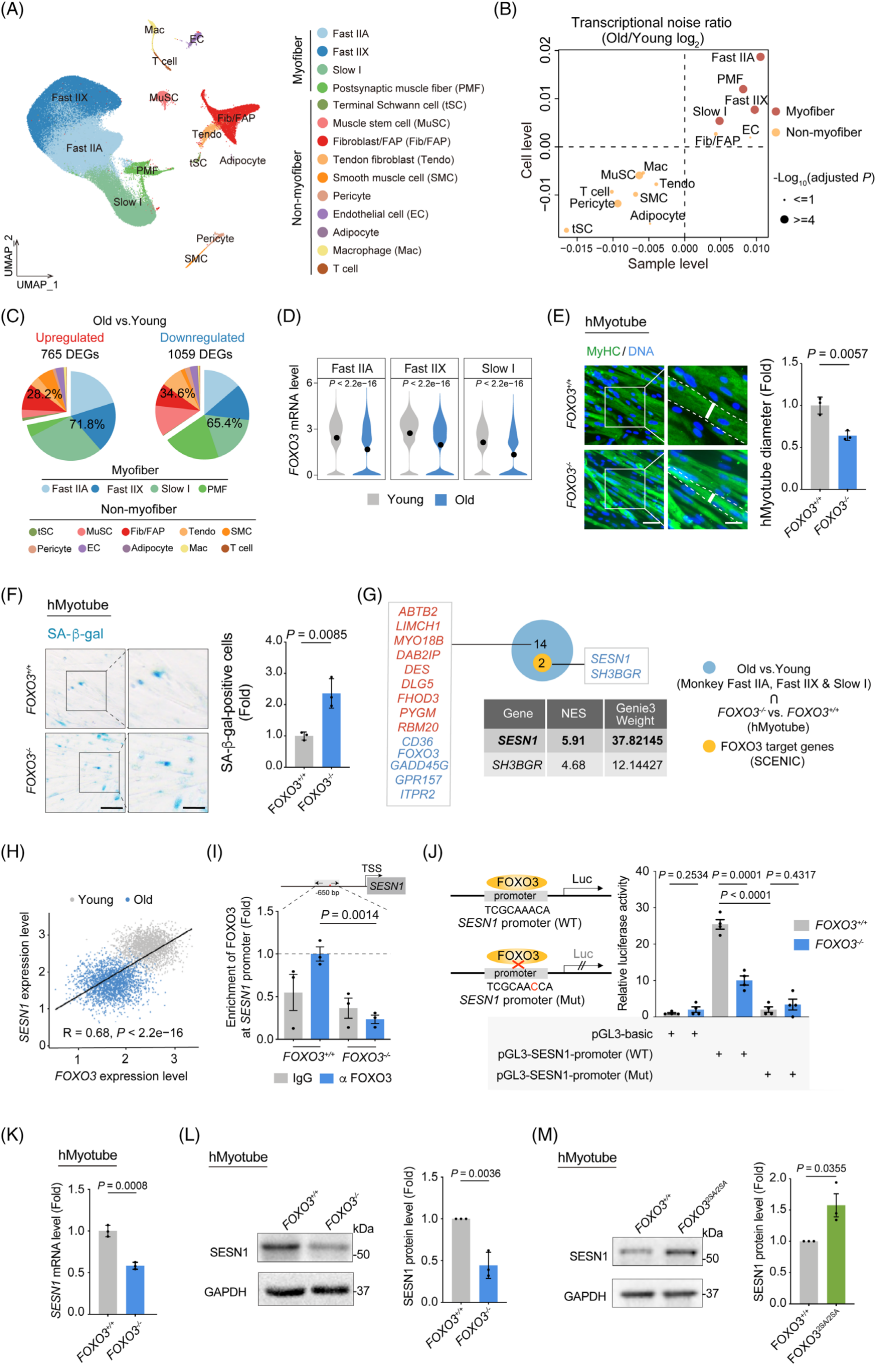
SESN1 is transactivated by FOXO3 in primate skeletal muscle. (A) Uniform manifold approximation and projection (UMAP) plot showing the 14 cell types of cynomolgus monkey skeletal muscle. Cells are annotated to the right. EC, endothelial cell; Fib/FAP, fibroblast/fibro‐adipogenic progenitor; Mac, macrophage; MuSC, muscle stem cell; PMF, postsynaptic muscle fibre; SMC, smooth muscle cell; Tendo, Tendon fibroblast; tSC, terminal Schwann cell. (B) Scatter plot showing the log_2_ ratio of transcriptional noise between old and young samples as calculated using sample averages and single cells on the *X* and *Y* axes (*n* = 16), respectively. (C) Pie charts showing the proportions of ageing‐related differentially expressed genes (DEGs) (adjusted *p* values < 0.05 and |LogFC| > 0.25) between old and young groups for each cell type. Left, upregulated DEGs; right, downregulated DEGs. (D) Violin plots showing *FOXO3* mRNA expression levels across Fast IIA, Fast IIX and Slow I cell types in cynomolgus monkey skeletal muscles between old and young groups. (E) MyHC immunofluorescence staining in *FOXO3* ^*+/+*^ and *FOXO3* ^*−/−*^ human myotubes (hMyotubes). Representative images are shown on the left. Scale bars, 50 and 25 μm (zoomed‐in image). Right, the diameters of the hMyotubes were quantified as fold changes (*FOXO3* ^*−/−*^ vs. *FOXO3* ^*+/+*^) and are presented as mean ± SEMs. *n* = 3 biological replicates. (F) Left, representative SA‐β‐gal staining images of *FOXO3* ^*+/+*^ and *FOXO3* ^*−/−*^ hMyotubes. Scale bars, 100 and 50 μm (zoomed‐in image). Right, the percentages of SA‐β‐gal‐positive hMyotubes were quantified as fold changes (*FOXO3* ^−/−^ vs. *FOXO3* ^+/+^) and are presented as mean ± SEMs. *n* = 3 biological replicates. (G) *SESN1* and *SH3BGR* were identified through analysis of overlapping genes between FOXO3 target genes and genes shared by aging associated DEGs in monkey myofibre and DEGs (*FOXO3* ^*−/−*^ vs. *FOXO3* ^*+/+*^) in hMyotubes. Table showing the weight information of *SESN1* and *SH3BGR* in FOXO3 regulated transcriptional network. (H) Scatter plot showing the relative expression levels of *FOXO3* and *SESN1*. Every 20 nuclei of myofibre in young and old groups were consolidated as a bin. The gene expression levels of each bin were calculated as the average expression of nuclei inside the specific bin. Each point marked in grey or blue represents a cell bin of young or old group, respectively. (I) ChIP‐qPCR analysis of the occupancy of FOXO3 on the *SESN1* promoter in myotubes using an anti‐FOXO3 antibody. The diagram above depicts the relative position of primers (arrows) used for ChIP‐qPCR. Data are presented as mean ± SEMs. *n* = 3 technical replicates for each group. (J) Dual luciferase reporter assay showing transcriptional activation of *SESN1* by FOXO3. *FOXO3* ^*+/+*^ and *FOXO3* ^*−/−*^ hMyotube progenitor cells were co‐transfected with plasmid expressing Renilla and vectors carrying wildtype (WT) or mutant (Mut) pGL3‐SESN1 promoter. (K) RT‐qPCR analysis showing the mRNA levels of *SESN1* in *FOXO3* ^*+/+*^ and *FOXO3^−/−^* hMyotubes. Data are presented as mean ± SEMs. *n* = 3 biological replicates. (L) Western blot analysis showing the protein levels of SESN1 in *FOXO3* ^*+/+*^ and *FOXO3* ^−/−^ hMyotubes. GAPDH was used as the loading control. Band intensities were quantified as fold changes (*FOXO3* ^*−/−*^ vs. *FOXO3* ^*+/+*^) and is presented as mean ± SEMs. *n* = 3 independent experiments. (M) Western blot analysis showing the protein levels of SESN1 in *FOXO3* ^*+/+*^ and *FOXO3* ^*2SA/2SA*^ hMyotubes. GAPDH was used as the loading control. Band intensities were quantified as fold changes (*FOXO3* ^*2SA/2SA*^ vs. *FOXO3* ^*+/+*^) and is presented as mean ± SEMs. *n* = 3 independent experiments. ChIP, chromatin immunoprecipitation; RT‐qPCR, real‐time quantitative PCR.

In a separate study, we recently established a single‐nucleus transcriptome atlas of primate skeletal muscle ageing^20^ (Figure 1A). Our data indicate a higher susceptibility of myofibres to ageing, as evidenced by higher transcriptional noise, and more DEGs in aged myofibre cells than those in non‐myofibre cells (Figure 1B,C). Remarkably, downregulation of *FOXO3*, a well‐known longevity gene,^20^, ^42^, ^43^ was identified in aged myofibres and contributed to primate skeletal muscle dyshomeostasis and degeneration (Figures 1D–F and S1A).

Next, we sought to investigate the molecular mechanism by which FOXO3 inactivation causes progressive muscle degeneration. Through motif analysis of DEGs shared between aged muscle (Old vs. Young) and FOXO3‐depleted human myotubes (hMyotubes) (*FOXO3* ^*−/−*^ vs. *FOXO3* ^*+/+*^), we discovered two genes, *SESN1* and *SH3BGR*, with potential FOXO3 binding sites 2 kb upstream of their transcription start sites (TSSs) (Figure 1G). Particularly, *SESN1* has a higher Genie3 weight (Figure 1G), along with its higher relevance with FOXO3 via co‐expression analysis, indicating its higher potential to be a target gene of FOXO3 (Figure 1H). To evaluate whether FOXO3 is capable of binding to the predicted site, we performed ChIP‐qPCR with an anti‐FOXO3 antibody. Using *FOXO3* ^*−/−*^ hMyotubes as a negative control, we observed specific binding between FOXO3 and the *SESN1* promoter in hMyotubes (Figure 1I). Subsequently, to query whether *SESN1* is directly activated by FOXO3, we cloned the promoter of *SESN1* upstream of the luciferase reporter and found that the promoter of *SESN1* was indeed transcriptionally activated by FOXO3 (Figure 1J). By contrast, we observed blunted *SESN1* promoter activity upon FOXO3 depletion as assessed by luciferase reporter assay (Figure 1J), and similarly, that a single nucleotide change (ACA to CCA) within the *SESN1* promoter reduced FOXO3 binding and substantially repressed luciferase reporter activity (Figure 1J). Consistently, SESN1 expression levels were markedly downregulated in *FOXO3* ^*−/−*^ hMyotubes relative to wildtype (WT) ones (Figure 1K,L). In contrast, we noticed elevated SESN1 expression levels of hMyotubes in which endogenous *FOXO3* was activated, and in which a constitutively active version of *FOXO3* was generated via gene editing‐based alanine substitution on two of the three classical phosphorylation sites^42^ (Figure 1M). Collectively, these observations support a role for FOXO3 in positively regulating *SESN1* transcription.

### 3.2 SESN1 mediates the geroprotective effects of FOXO3 on primate skeletal muscle

**FIGURE 2:**
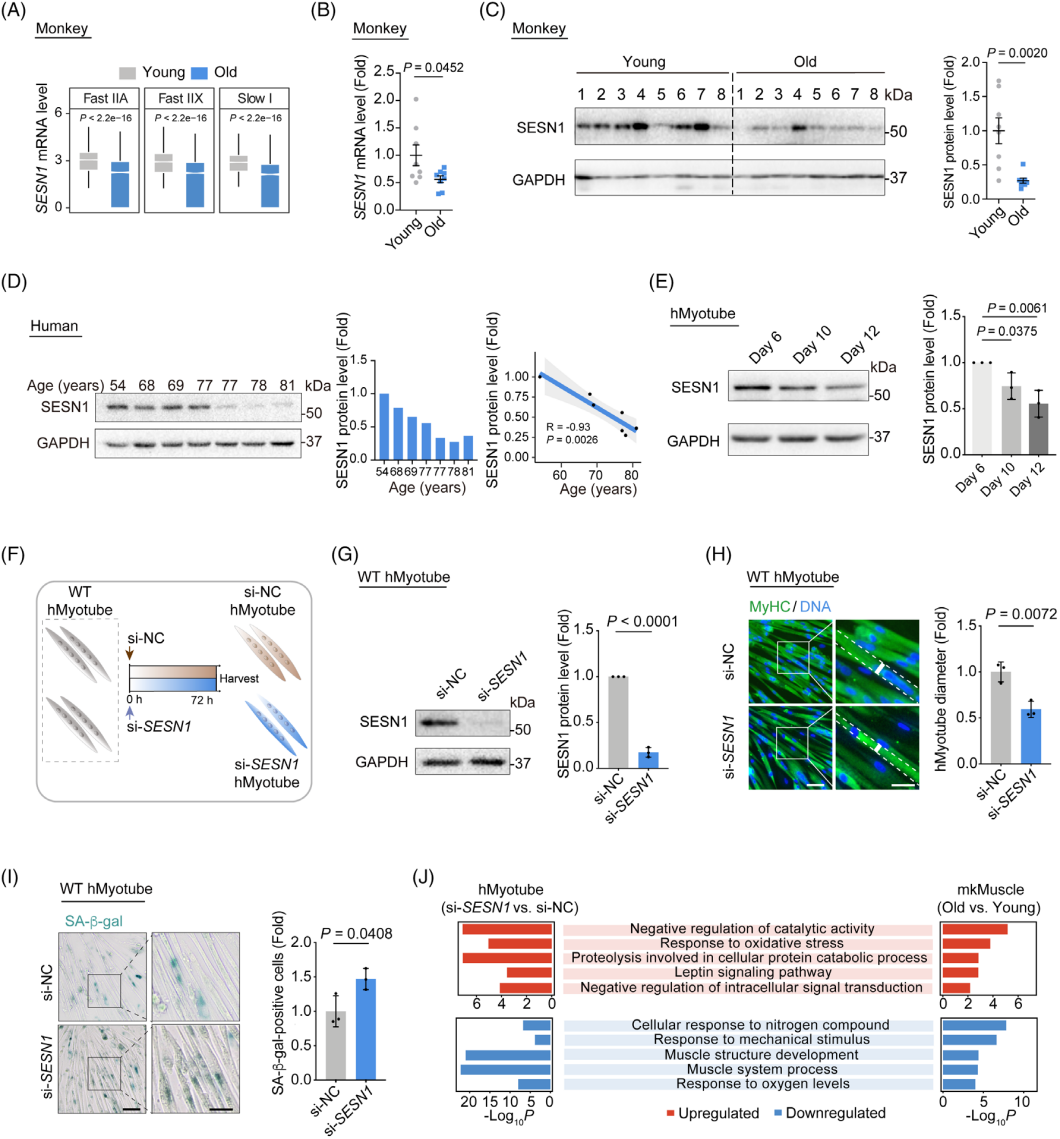
SESN1 exerts a key protective role in counteracting primate skeletal muscle ageing. (A) Box plots showing *SESN1* mRNA expression levels across Fast IIA, Fast IIX and Slow I cell types in monkey myofibres between old and young groups. (B) RT‐qPCR analysis showing the *SESN1* mRNA expression changes in young and old monkey skeletal muscles. *SESN1* mRNA levels were quantified as fold changes (old vs. young) and are presented as mean ± SEMs. *n* = 8 monkeys for each group. (C) Western blot analysis and band intensity quantification of SESN1 protein levels in young and old monkey skeletal muscle samples. Data were quantified as fold changes (old vs. young) and are presented as mean ± SEMs. *n* = 8 monkeys for each group. (D) Left, western blot analysis and band intensity quantification of SESN1 protein levels in human skeletal muscle samples. GAPDH was used as the loading control. Middle, bar plot showing the relative expression of SESN1 protein levels for each individual. Right, the negative correlation of relative SESN1 protein levels in skeletal muscle across different ages. The shadow indicates the 0.95 confidence interval around smooth. *n* = 7 donors. (E) Western blot analysis showing the protein levels of SESN1 in WT hMyotubes after a prolonged‐culture for 6, 10 and 12 days. GAPDH was used as the loading control. Band intensities were quantified as fold changes in hMyotubes at Day 10 or Day 12 versus at Day 6 and is presented as mean ± SEMs. *n* = 3 independent experiments. (F) Schematic showing the method used to generate *SESN1*‐knockdown hMyotubes. (G) Western blot analysis showing the protein levels of SESN1 in hMyotubes transfected with si‐NC or si‐*SESN1*. GAPDH was used as the loading control. Band intensities were quantified as fold changes (si‐*SESN1* vs. si‐NC) and is presented as mean ± SEMs. *n* = 3 independent experiments. (H) MyHC immunofluorescence staining of the hMyotubes transfected with si‐NC or si‐*SESN1*. Representative images are shown on the left. Scale bars, 50 and 25 μm (zoomed‐in image). Right, the diameters of the hMyotubes were quantified as fold changes (si‐*SESN1* vs. si‐NC) and are presented as mean ± SEMs. *n* = 3 biological replicates. (I) Representative images of SA‐β‐gal‐positive hMyotubes transfected with si‐NC or si‐*SESN1*. Representative images are shown on the left. Scale bars, 100 and 50 μm (zoomed‐in image). Data were quantified as fold changes (si‐*SESN1* vs. si‐NC) and are presented as mean ± SEMs on the right. *n* = 3 biological replicates. (J) GO terms shared by DEGs between old and young monkey skeletal muscle (mkMuscle) and DEGs between si‐*SESN1* and si‐NC hMyotubes. DEG, differentially expressed gene; GO, gene ontology; RT‐qPCR, real‐time quantitative PCR; WT, wildtype.

In line with the downregulation of FOXO3 in aged primate skeletal muscles, we also found a consistent downregulation of *SESN1* mRNA and protein expression levels in almost all aged cynomolgus monkey myofibre cells and in skeletal muscles as an entity (Figure 2A–C). More strikingly, we observed an age‐dependent decline of SESN1 protein in human skeletal muscle, along with decreased expression in prolonged‐cultured senescent hMyotubes (Figure 2D,E). Altogether, these findings demonstrate that inactivation of the FOXO3‐SESN1 axis is a pronounced molecular characteristic of primate skeletal muscle ageing.

We next asked whether SESN1 mediates the geroprotective effect of FOXO3 in primate skeletal muscle. Indeed, we found that *SESN1* knockdown mimicked the human myotube ageing phenotypes we had observed in FOXO3‐deficient hMyotubes, as evidenced by a decrease in human myotube diameter and concomitant elevation of SA‐β‐gal activity (Figure 2F–I). In addition, a subset of DEGs overlapped between *FOXO3* ^*−/−*^ and *SESN1*‐knockdown hMyotubes, including 87 upregulated genes and 49 downregulated genes (i.e., muscle structure‐associated genes, *MYBPC2*, *MYL1* and *PKP2*) (Figure S1B–E). Furthermore, when we compared transcriptomes of SESN1‐deficient hMyotubes and ageing‐related DEGs in aged muscle (Table S1), we found that shared upregulated genes were associated with catabolic proteolysis and oxidative stress, whereas shared downregulated genes were associated with muscle functions (i.e., muscle structure development) (Figure 2J).

**FIGURE 3:**
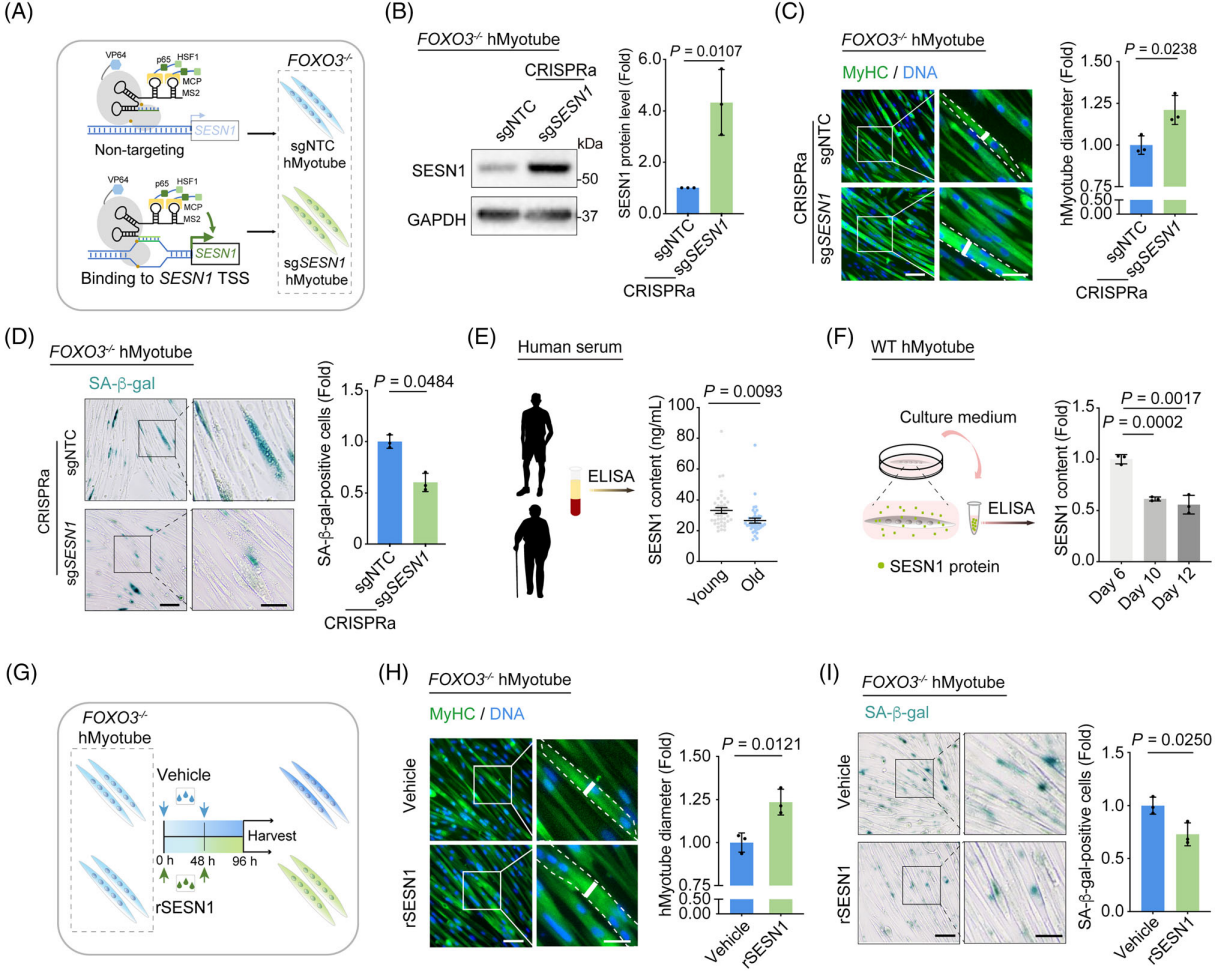
Activation of *SESN1* gene expression and supplementation of recombinant SESN1 protein attenuate human myotube senescence. (A) Schematic showing the method used to activate the endogenous *SESN1* in *FOXO3* ^−/−^ hMyotubes by CRISPR/dCas9‐mediated transcriptional activation system. (B) Western blot analysis showing the protein levels of SESN1 in hMyotubes upon the CRISPR/dCas9‐mediated activation of *SESN1* gene expression. GAPDH was used as the loading control. Band intensities were quantified as fold changes (CRISPRa‐sg*SESN1* vs. CRISPRa‐sgNTC) and is presented as mean ± SEMs. n = 3 independent experiments. (C) MyHC immunofluorescence staining of the hMyotubes upon the CRISPR/dCas9‐mediated activation of *SESN1* gene expression. Representative images are shown on the left. Scale bars, 50 and 25 μm (zoomed‐in image). Right, the diameters of the hMyotubes were quantified as fold changes (CRISPRa‐sg*SESN1* vs. CRISPRa‐sgNTC) and are presented as mean ± SEMs. *n* = 3 biological replicates. (D) SA‐β‐gal‐positive cells of the hMyotubes upon the CRISPR/dCas9‐mediated activation of *SESN1* gene expression. Representative images are shown on the left. Scale bars, 100 and 50 μm (zoomed‐in image). Data were quantified as fold changes (CRISPRa‐sg*SESN1* vs. CRISPRa‐sgNTC) and are presented as mean ± SEMs on the right. *n* = 3 biological replicates. (E) ELISA analysis showing the SESN1 content in the sera from young and old individuals. Data are presented as mean ± SEMs. n = 40 donors per group. (F) ELISA analysis showing the SESN1 content in conditioned medium of WT hMyotubes after a prolonged‐culture for 6, 10 and 12 days. Data are presented as mean ± SEMs. *n* = 3 biological replicates. (G) Schematic showing the method of recombinant SESN1 (rSESN1) protein treatment in *FOXO3* ^*−/−*^ hMyotubes. (H) MyHC immunofluorescence staining of the *FOXO3* ^*−/−*^ hMyotubes treated with Vehicle or rSESN1 protein. Representative images are shown on the left. Scale bars, 50 and 25 μm (zoomed‐in image). Right, the diameters of the hMyotubes were quantified as fold changes (rSESN1 vs. Vehicle) and are presented as mean ± SEMs. *n* = 3 biological replicates. (I) Representative images of SA‐β‐gal‐positive cells of *FOXO3* ^*−/−*^ hMyotubes treated with Vehicle or rSESN1 protein. Representative images are shown on the left. Scale bars, 100 and 50 μm (zoomed‐in image). Data were quantified as fold changes (rSESN1 vs. Vehicle) and are presented as mean ± SEMs on the right. *n* = 3 biological replicates. ELISA, enzyme‐linked immunosorbent assay; WT, wildtype.

Given these overlaps, we wondered whether the ageing phenotypes in FOXO3‐deficient hMyotubes could be rescued by overexpression of SESN1. To this end, we induced endogenous SESN1 expression using the CRISPR‐dCas9 transcriptional activation system (Figure 3A,B). As expected, we observed that ageing features in the *FOXO3* ^*−/−*^ hMyotubes were mitigated upon induction of SESN1 expression, as demonstrated by increased human myotube diameter and concomitant reduction of SA‐β‐gal activity (Figure 3C,D). At gene expression levels, SESN1‐rescued genes were associated with the relieved defects in FOXO3‐depleted hMyotubes (Figure S1F–H and Table S1). Taken together, these data suggest that SESN1, as a major downstream effector of FOXO3, plays a pivotal role in safeguarding skeletal muscle against ageing.

### 3.3 SESN1 serves as a protective secretory factor against human myotube senescence

Intriguingly, SESN1 was recently recognized to be a secreted protein.^44^ Given our data showing that its expression was downregulated in skeletal muscle (the largest organ in the human body) with age, we next asked whether its blood level in human individuals decreases with age. As expected, we found that SESN1 levels were significantly lower in serum of older human individuals than in younger individuals (Figure 3E). Consistently, SESN1 levels were also reduced in conditioned medium of prolonged‐cultured senescent hMyotubes (Figure 3F).

Given these findings, we asked if exogenous supplementation of recombinant SESN1 protein could restore ageing‐associated compromised skeletal muscle function. Impressively, in *FOXO3* ^*−/−*^ hMyotubes, we found that treatment with recombinant SESN1 protein at a concentration of 0.5 μg/mL rescued the senescent phenotypes, as evidenced by increased myotube diameter and reduced SA‐β‐gal activity (Figure 3G–I). These results indicate that SESN1 serves as a secreted factor that protects against skeletal muscle ageing, suggesting directions for exploring SESN1‐based intervention strategies for delaying skeletal muscle ageing.

### 3.4 Recombinant SESN1 protein boosts muscle regeneration *in vivo*

**FIGURE 4:**
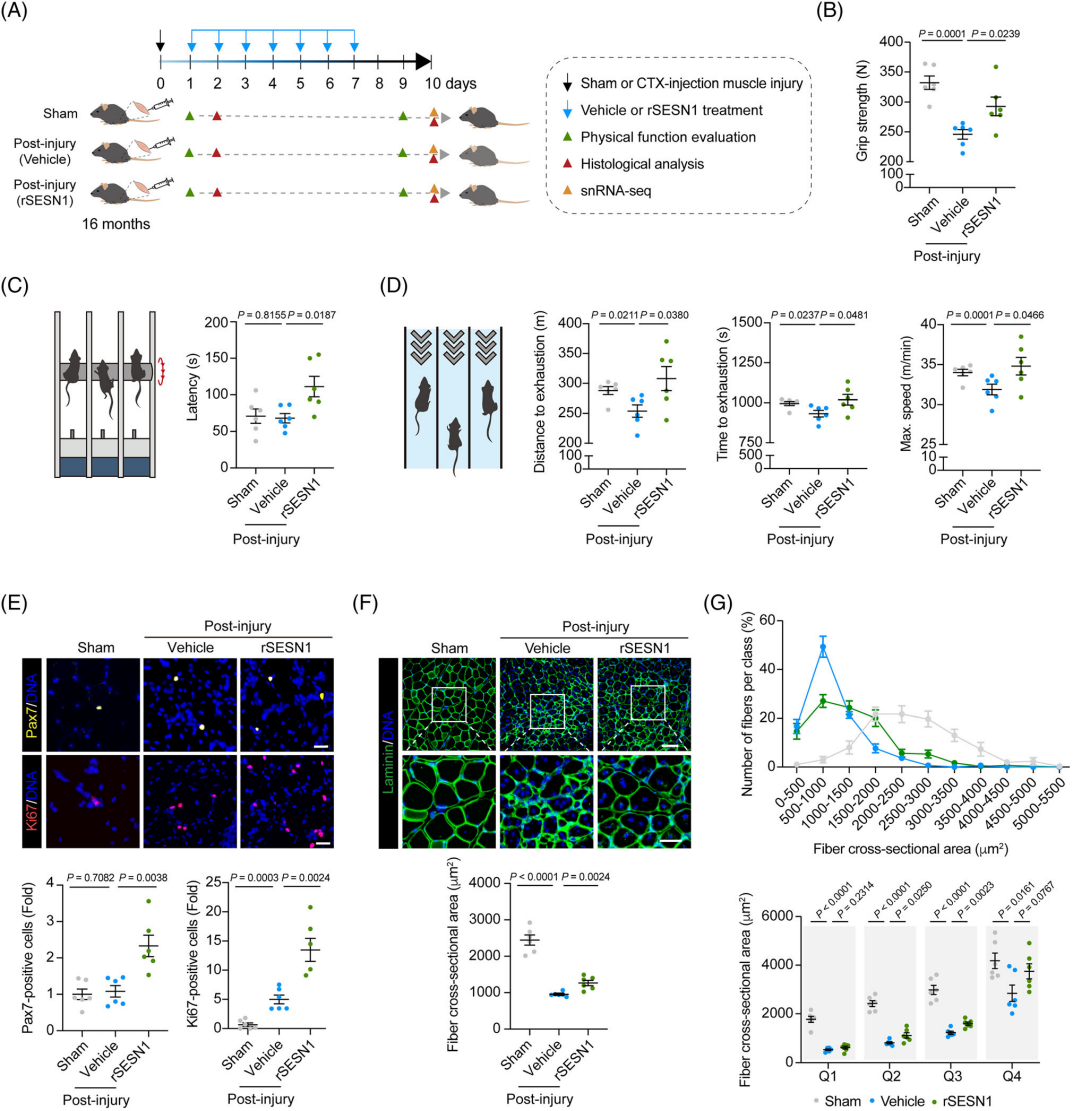
Recombinant SESN1 protein facilitates skeletal muscle regeneration *in vivo*. (A) Schematic diagram showing the experimental designs and time course of the Sham, cardiotoxin (CTX)‐induced mouse skeletal muscle injury control (post‐injury‐Vehicle) or recombinant SESN1 protein treatment (post‐injury‐rSESN1). (B) Grip test strength evaluation in mice of Sham, post‐injury‐Vehicle and post‐injury‐SESN1 groups at Day 9. Data were quantified and are presented as mean ± SEMs on the right. *n* = 6 mice for each group. (C) Grid‐hanging capacity evaluation in Sham, post‐injury‐Vehicle and post‐injury‐rSESN1 groups at Day 9. Data were quantified and are presented as mean ± SEMs on the right. *n* = 6 mice for each group. (D) Distance and time to exhaustion and maximal speed analyses in mice of Sham, post‐injury‐Vehicle and post‐injury‐rSESN1 groups at Day 9. Data were quantified and are presented as mean ± SEMs. *n* = 6 mice for each group. (E) Top, the representative immunofluorescence staining images of Pax7 and Ki67 in skeletal muscles of Sham, post‐injury‐Vehicle and post‐injury‐rSESN1 groups at Day 10. Scale bars, 25 μm. Bottom, the relative Pax7‐ and Ki67‐positive cells were quantified as fold changes and are presented as mean ± SEMs. *n* = 6 mice for each group. (F) Muscle fibre cross‐sectional area in mice of Sham, post‐injury‐Vehicle and post‐injury‐rSESN1 groups at Day 10. Top, representative immunofluorescence staining images of cross‐sectional area of fibres, which were labelled by Laminin antibody in green. Scale bars, 100 μm and 50 μm (zoomed‐in image). Bottom, the cross‐sectional area of fibres was quantified and is presented as mean ± SEMs. *n* = 6 mice for each group. (G) Top, frequency distribution of muscle fibre cross‐section area in Sham, post‐injury‐Vehicle and post‐injury‐rSESN1 groups at Day 10. Bottom, the calculated lower quartile (Q1), the middle quartile (Q2; median of the data), the upper quartile (Q3) and the maximum value in the range (Q4) of the fibre size distribution. *n* = 6 mice for each group.

Since senescent cells accumulate with age and hamper muscle regeneration, declined regeneration capability associated with age‐accumulated damage is a hallmark feature of the elderly.^45^, ^46^ Thus, we next asked whether recombinant SESN1 protein treatment harbours a beneficial effect on skeletal muscle regeneration *in vivo*. To this end, we treated aged mice (16 months old) with CTX to obtain a classic muscle injury and regeneration model,^47^, ^48^ and subsequently administrated daily recombinant SESN1 protein for 7 days (Figures 4A and S2A,B). Indeed, our data showed that administration of recombinant SESN1 protein, relative to Vehicle‐treated control, improved grip strength and physical endurance, as reflected by prolonged grid‐hanging time, as well as increased maximal running time and running distance (Figure 4B–D), indicating that exogenous SESN1 protein supplementation substantially enhances athletic ability after muscle injury in mice.

As quiescent muscle stem cells (MuSCs) become activated and proliferate to generate myofibres in response to physiological or pathological stimuli (e.g., toxin drug‐induced injury),^17^ we next examined the changes of MuSCs and found an elevated proportion of Pax7‐positive cells and Ki67‐positive mitotic cells after SESN1 protein treatment (Figure 4E). Furthermore, a shift towards larger embryonic MHC (eMHC)‐positive fibres, a classic trait for skeletal muscle regeneration,^17^, ^49^ was also observed in the SESN1‐supplemented muscle (Figure S2C). Most importantly, when we measured the cross‐sectional area of skeletal muscle fibres, we noticed a marked increment in the diameter of myofibres in SESN1‐treated mice (Figure 4F,G). Overall, these findings suggest recombinant SESN1 protein treatment in mice effectively enhance generation ability after muscle injury.

### 3.5 Single‐nucleus profiling resolves the pro‐regenerative transcriptomic signatures underlying recombinant SESN1 protein supplement

**FIGURE 5:**
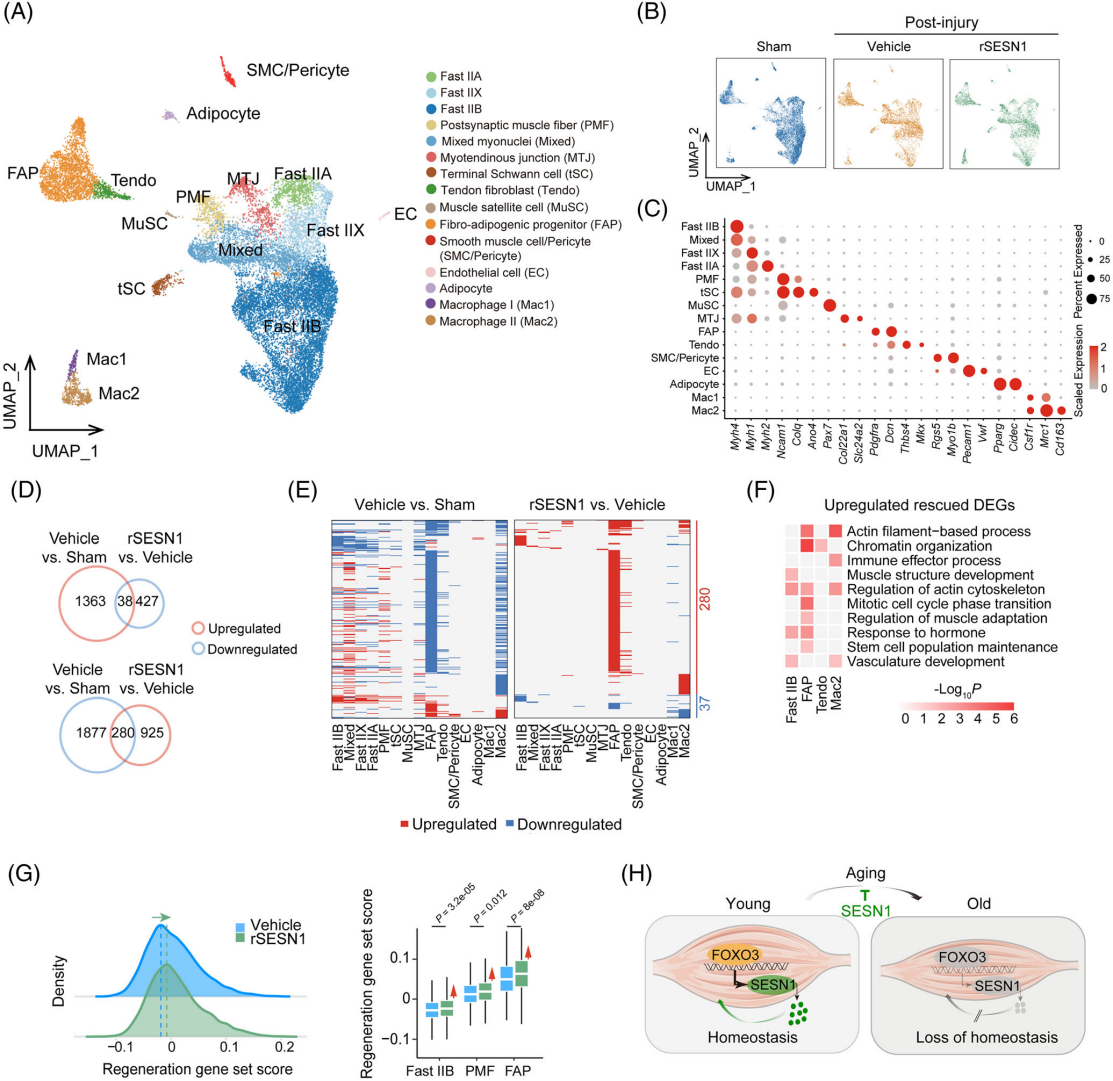
Single‐nucleus transcriptomic analysis of skeletal muscle from mice treated with recombinant SESN1 protein. (A) UMAP plot showing mouse skeletal muscle cell types by single‐nucleus RNA‐sequencing. Cells are coloured by types and annotated to the right. (B) UMAP plot showing the cell distribution in Sham, post‐injury‐Vehicle and post‐injury‐rSESN1 groups. (C) Dot plot showing the expression of representative marker genes for each cell type in mouse skeletal muscle. (D) Venn diagram showing the number and percentage of upregulated (top) and downregulated (bottom) DEGs induced by CTX treatment and rescued by rSESN1 protein administration. Rescued DEGs exhibiting the opposite changes upon CTX‐induced injury and rSESN1 protein treatment. (E) Heatmaps showing the distribution of DEGs across different cell types between post‐injury‐Vehicle and Sham groups, and between post‐injury‐rSESN1 and post‐injury‐Vehicle groups, respectively. Each row represents one gene, and each column represents one cell type. (F) GO term and pathway enrichment analysis for upregulated rescued DEGs. The colour key from white to red indicates ‐Log_10_ (*p* value) from low to high. (G) Left, ridge map showing the global distribution density of regeneration‐related gene set score for DEGs in post‐injury‐Vehicle and post‐injury‐rSESN1 groups treated mouse skeletal muscle. The corresponding dashed line represents the peak position of each group. Right, box plot showing the regeneration‐related gene set score across Fast IIB, PMF and FAP types of mouse muscle in post‐injury‐Vehicle and post‐injury‐rSESN1 groups. (H) A working model for FOXO3‐SESN1 axis in the homeostatic regulation during skeletal muscle aging. CTX, cardiotoxin; DEG, differentially expressed gene; GO, gene ontology; UMAP, uniform manifold approximation and projection.

To resolve single‐cell transcriptional changes underlying the SESN1‐driven regenerative response after injury, we performed single‐nucleus RNA sequencing (snRNA‐seq) (Figure 5A–C). After stringent filtration, we retained 19,044 qualified single nuclei for downstream analyses. By using unbiased clustering and uniform manifold approximation and projection analysis, and based on canonical cell markers, we identified 15 nuclear profiles with distinct transcriptomic signatures (Figure 5A–C), including five types of myonuclei from myofibres (Fast IIA, Fast IIX, Fast IIB, mixed myonuclei [Mixed] and postsynaptic muscle fibre [PMF]), as well as a total of 10 non‐myonuclear cell types (Myotendinous junction [MTJ], terminal Schwann cell [tSC], tendon fibroblast [Tendo], MuSC, fibro‐adipogenic progenitor [FAP], smooth muscle cell/pericyte [SMC/Pericyte], endothelial cell [EC] and adipocyte) and two immune cell types (macrophage I [Mac1] and macrophage II [Mac2]).

Next, we investigated cell type‐specific molecular alterations affected by recombinant SESN1 protein administration across different cell types. Compared to the Vehicle‐treated group, snRNA‐seq analysis demonstrated that recombinant SESN1 protein supplementation reversed the injury‐related gene expression profiling and resulted in a global pro‐regenerative transcriptomic signature (Figure 5D–G). Specific features included enhanced expression of a panel of genes related to muscle structure development in Fast IIB myofibre, and genes involved in mitotic cell cycle in FAP, a muscle‐resident mesenchymal stromal cell responsible for muscle regeneration (Figure 5D–F).^50^ Importantly, the scores of regeneration‐related genes were higher in SESN1‐treated skeletal muscle and particularly in Fast IIB myofibre and FAP relative to Vehicle‐treated counterparts (Figure 5G). Taken together, these results suggest that recombinant SESN1 protein has therapeutic potential for promoting muscle regeneration and delaying muscle ageing.

## 4 DISCUSSION

Age‐related decrease in skeletal muscle mass and strength is a hallmark of sarcopenia.^51^ As such, this decline impairs physical performance and increases metabolic disease risk in the elders. Here, we identified the FOXO3‐SESN1 axis as a gatekeeper mechanism protecting against primate skeletal muscle ageing. Notably, SESN1 levels were reduced in the serum of elderly human individuals, implying that SESN1 could function as a potent circulating biomarker to predict progressive skeletal muscle atrophy. More importantly, we propose that recombinant SESN1 protein may have potential as a therapeutic agent capable of attenuating myofibre senescence and promoting muscle regeneration (Figure 5H). These novel findings identify SESN1 as a robust mediator antagonizing myofibre senescence, paving the way for development of novel diagnostics and intervention therapies for human skeletal muscle ageing.

In this study, we identified SESN1 as a major effector downstream of FOXO3 that exerts a potent protective role against skeletal muscle ageing. In broadly related work, FOXO1, another member of the FOXO family, was reported to directly regulate expression levels of the SESN1 homologue SESN3 in human tumour cells,^52^ supporting our observations and suggesting a conserved mechanism by which FOXO proteins transcriptionally regulate members of the Sestrin family.

To be noted, we detected diminished secretion of SESN1 in the senescent human myotube model and lower SESN1 protein level in serum from human elderly. Interestingly, a recent study reported decreased SESN1 protein levels in serum of the elderly with sarcopenia relative to the elderly not afflicted with sarcopenia.^44^ These clinical observations, together with our data, suggest that reduced secretion of myokine SESN1 may represent a potential predictive biomarker of muscle ageing, and an advance warning sign of sarcopenia. Most strikingly, we show that exogenous supplementation of recombinant SESN1 protein alleviated human myotube senescence in vitro and boosted muscle repair and regeneration *in vivo*.^17^ Although resistance exercise and nutritional supplementation are known to augment muscle mass accretion and improve muscle strength, these intervention strategies are less realistic in the elderly population, most of whom have massive impairments in physical performance and anorexia.^1^, ^53^, ^54^ On the other hand, beneficial effects of hormone replacement therapy on muscle atrophy, such as pharmacological interventions with testosterone and growth hormone in patients with low‐baseline levels, remain controversial^1^, ^3^ and are associated with cardiovascular risks and other safety issues,^3^ presenting significant challenges for clinical translation. Therefore, there are no effective and safe targeted interventions to mitigate or reverse age‐related muscle loss. Given the high unmet need, the data showing that exogenous supplementation of myokine SESN1 alleviating ageing‐related muscle degeneration alongside promoting the muscle regeneration and repair in preclinical models are encouraging. Since human recombinant protein can be produced in large quantities with high quality, this study paves the way for exploring a myokine‐based therapeutic strategy with more attractive safety and convenience features.

In conclusion, based on our previously established single‐nucleus transcriptomic landscape of primate skeletal muscle ageing,^20^ we explored the potential of SESN1 as a novel biomarker of human skeletal muscle ageing. In functional models, we also demonstrate that treatment with recombinant SESN1 protein inhibits myofibre ageing and promotes its regeneration and repair. Our approach supports further explorations of additional diagnostic biomarkers of skeletal muscle ageing and development of novel therapeutic interventions to treat ageing‐associated muscle diseases.

## AUTHOR CONTRIBUTIONS

Si Wang, Guang‐Hui Liu, Jing Qu and Yang Yu conceptualized this project and supervised the overall experiments. Ying Jing performed the phenotypic and mechanistic analyses. Yuesheng Zuo performed bioinformatics analyses of the snRNA‐seq and bulk RNA‐seq data. Huifang Hu performed the ChIP‐qPCR assay. Si Wang, Guang‐Hui Liu, Jing Qu, Yang Yu, Ying Jing, Yuesheng Zuo, Liang Sun, Zheng‐Rong Yu, Shuai Ma, Huifang Hu, Qian Zhao, Daoyuan Huang, Weiqi Zhang and Juan Carlos Izpisua Belmonte performed manuscript writing, review and editing. All authors reviewed the manuscript.

## CONFLICT OF INTEREST STATEMENT

The authors declare no conflict of interest.

## Supporting information


**FIGURE S1.** Transcriptional profiling upon knockdown or CRISPR/dCas9‐mediated activation of *SESN1* in human myotubes.

**FIGURE S2.** Physical activity and histochemistry analysis of injury muscle administrated with recombinant SESN1 protein.

**TABLE S1.** Differentially expressed genes of bulk RNA‐seq and snRNA‐seq datasets.

**TABLE S2.** The marker genes of each cell type in mouse skeletal muscle.

**TABLE S3.** Antibodies used in this study.

**TABLE S4.** The sequences for primers and siRNA used in this study.

## Data Availability

The accession numbers for the raw snRNA‐seq data and bulk RNA‐seq data reported in this paper are GSA: CRA000254 and HRA002774.
