# Supplementary figures and images for: SESN1 is a FOXO3 effector that counteracts human skeletal muscle ageing
Source: Cell Prolif. 2023 May 17;56(5):e13455. doi: 10.1111/cpr.13455 (PMC10212707; doi:10.1111/cpr.13455)

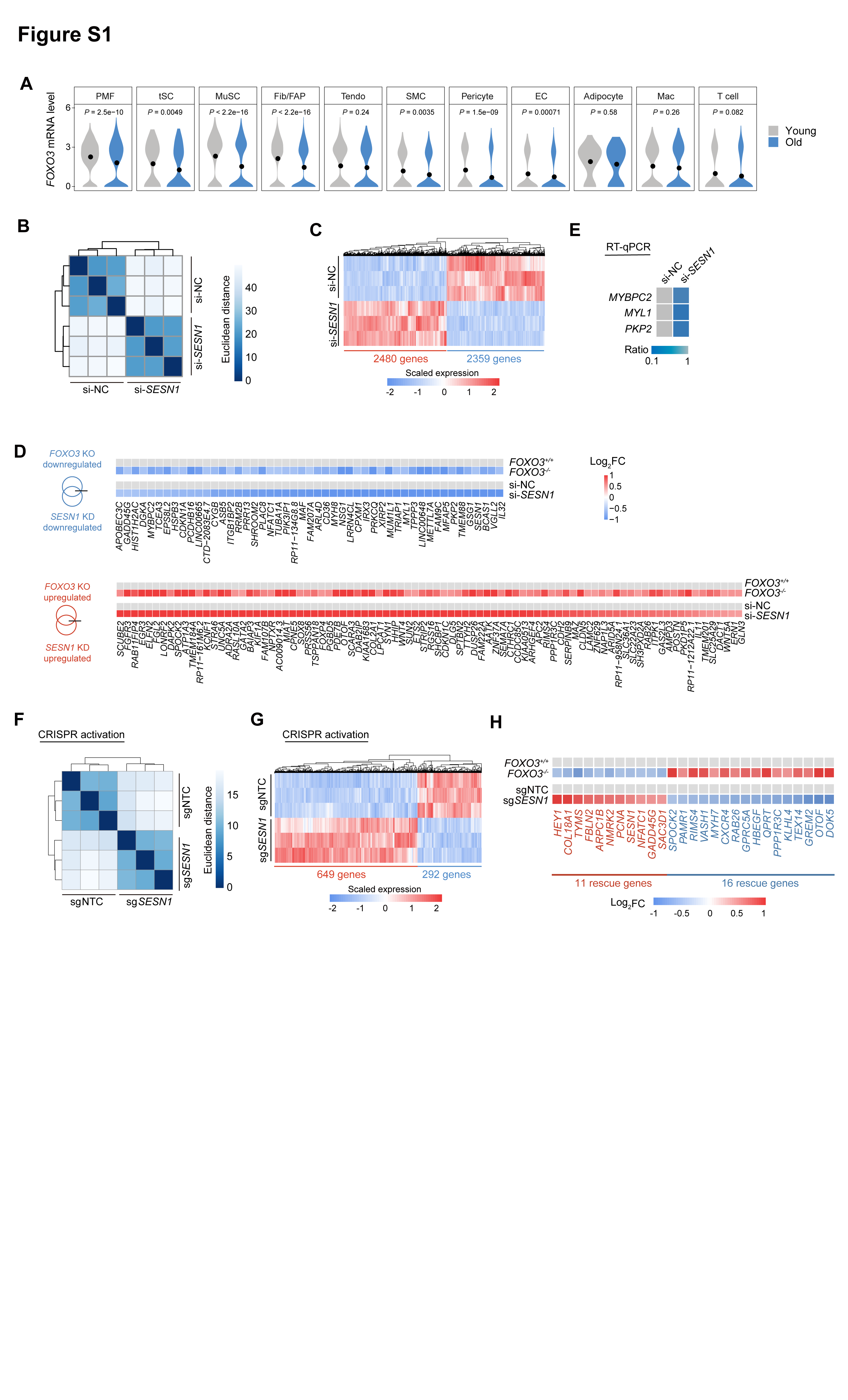

Supplement: Supplementary file 1 — FIGURE S1. Transcriptional profiling upon knockdown or CRISPR/dCas9‐mediated activation of SESN1 in human myotubes. [file CPR-56-e13455-s001.tif]

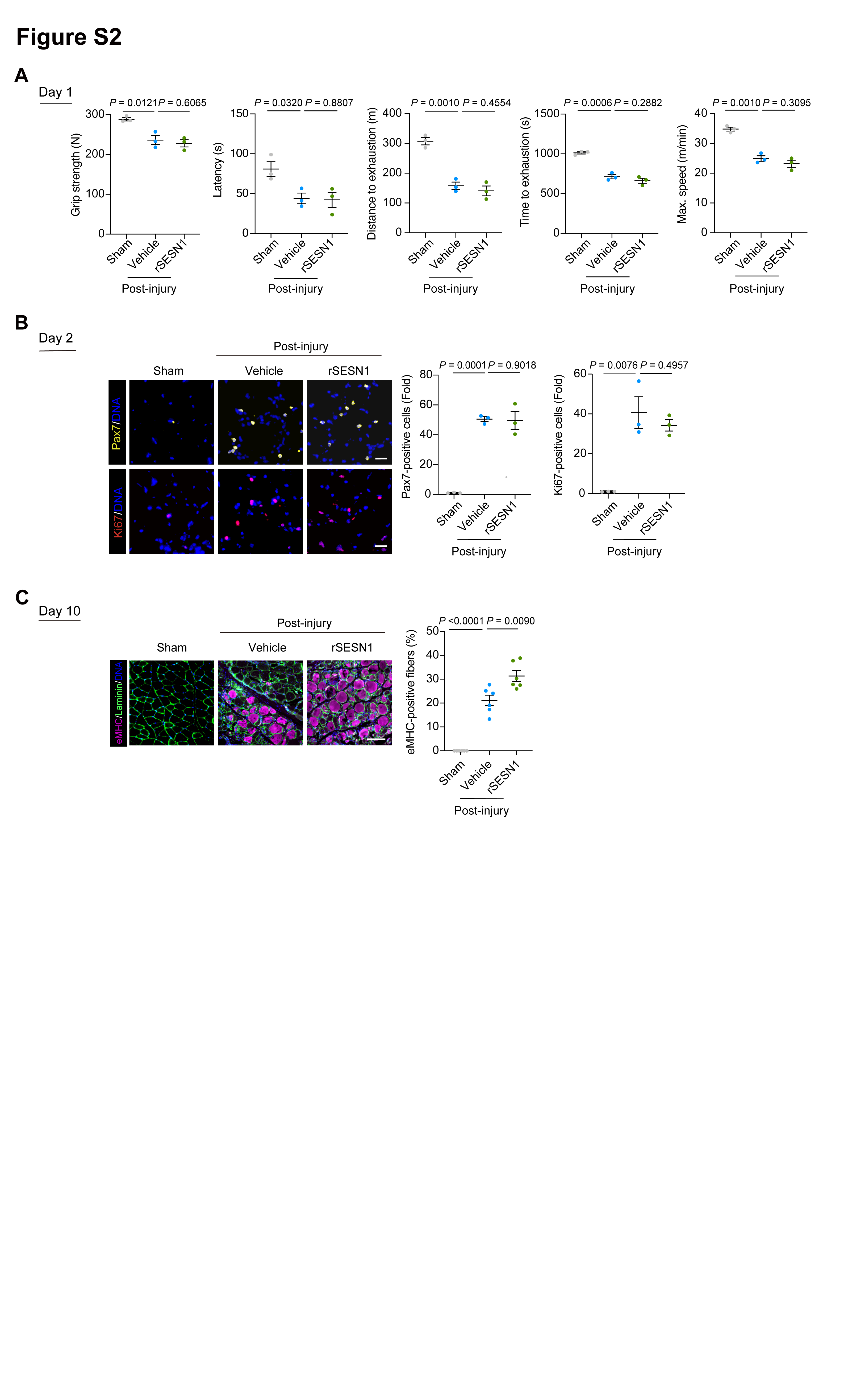

Supplement: Supplementary file 2 — FIGURE S2. Physical activity and histochemistry analysis of injury muscle administrated with recombinant SESN1 protein. [file CPR-56-e13455-s006.tif]
